# Characterization of Delta-7 Alkenone Desaturase in Haptophyte *Gephyrocapsa huxleyi* Through Heterologous Expression in *Tisochrysis lutea*

**DOI:** 10.1007/s10126-025-10427-y

**Published:** 2025-02-08

**Authors:** Kohei Yoneda, Chinatsu Kobayashi, Hiroya Araie, Rikuri Morita, Ryuhei Harada, Yasuteru Shigeta, Hirotoshi Endo, Yoshiaki Maeda, Iwane Suzuki

**Affiliations:** 1 Institute of Life and Environmental Sciences, University of Tsukuba, 1-1-1 Tennodai, Tsukuba, Ibaraki 305-8572 Japan; 2 Graduate School of Science and Technology, University of Tsukuba, 1-1-1 Tennodai, Tsukuba, Ibaraki 305-8572 Japan; 3 Department of Biosciences, College of Science and Technology, Kanto Gakuin University, Mutsuura-Higashi, Kanazawa-Ku, Yokohama, Kanagawa 236-8501 Japan; 4 Center for Computational Sciences, University of Tsukuba, 1-1-1 Tennodai, Tsukuba, Ibaraki 305-8577 Japan; 5 National Institute of Technology, Tsuruoka College, 104 Sawada, Inooka, Tsuruoka, Yamagata 997-8511 Japan

**Keywords:** *Gephyrocapsa huxleyi*, Alkenones, Desaturase, Transformation, Heterologous expression, *Tisochrysis lutea*

## Abstract

The marine haptophyte *Gephyrocapsa huxleyi* is an ecologically and geochemically important phytoplankton due to its contribution to the global carbon cycle and its ability to biosynthesize certain alkenones. These alkenones are long-chain alkyl ketones with two to four *trans*-type double bonds. The genes encoding alkenone desaturase in *G. huxleyi* have not been experimentally characterized so far, partly due to the difficulty of inducing genetic transformation in *G. huxleyi*. Therefore, we introduced the putative alkenone delta-7 desaturase of *G. huxleyi* (designated “DesT”) to the transformable and alkenone-producing haptophyte *Tisochrysis lutea*. We found two types of coding sequences for DesT, which are probably derived from the expression products of different alleles, and designated them “DesT-1” and “DesT-2.” The ratio of C_37:3_ to C_37:2_ methyl alkenone in the DesT-1 transformant was significantly higher than that in the mock strain that expressed only the hygromycin resistance gene, suggesting that DesT-1 was an alkenone delta-7 desaturase in *G. huxleyi*. In the protein structure, a tunnel where a substrate alkenone penetrates was predicted to be located around the histidine box of DesT, and hydrophilic and hydrophobic amino acids were respectively located at the proximal (near side to the histidine box) and distal ends of the tunnel. This is the first study to conduct experimental characterization of the alkenone metabolism-related gene in *G. huxleyi*. The heterologous expression system using *T. lutea* paves the way for further characterization of the alkenone metabolism-related genes in less transformable haptophytes.

**Supplementary Information:**

The online version contains supplementary material available at 10.1007/s10126-025-10427-y.

## Introduction

The marine haptophyte *Gephyrocapsa huxleyi* is a cosmopolitan phytoplankton that is distributed from the equator to the polar ocean and often produces huge blooms in early to midsummer (Brown and Yoder 1994; Harada et al. 2012). This alga produces coccolith scales made of calcium carbonate, and the scales sink to the bottom of the ocean after the bloom. This biological pump is considered to contribute greatly to the global carbon cycle (Iglesias-Rodríguez et al. 2002). In addition, *G. huxleyi* produces characteristic lipids called alkenones. These alkenones are long-chain (C_37–40_) unsaturated alkyl ketones with two to four *trans*-type double bonds and are produced in limited species of haptophytes, especially those belonging to Isochrysidales, such as *G. huxleyi*, *G. oceanica*, *Isochrysis galbana*, *Tisochrysis lutea*, and *Ruttnera lamellosa* (Theroux et al. 2010; Araie et al. 2018). The alkenones are thought to be accumulated as storage compounds in these haptophytes (Epstein et al. 2001; Tsuji et al. 2015; Bakku et al. 2018). Those found in marine sediments have been utilized for paleotemperature estimation (Conte et al. 2006) because the degree of unsaturation in an alkenone molecule is altered by the growth temperature of the alkenone-producing haptophyte (Conte et al. 1998). In addition, these alkenone molecules have attracted attention as feedstock for biofuels (Yamane et al. 2013; O’Neil et al. 2015). However, despite their importance in biogeochemistry and biofuel production, the biosynthetic and metabolic pathways of alkenones are not well understood.

The alkenones have a long alkyl chain, which is thought to be biosynthesized from the condensation of malonyl-CoA-like fatty acids (Rontani et al. 2006; Zheng et al. 2016). The alkenone molecules have at least two *trans*-type double bonds at the delta-14 and delta-21 positions. These two double bonds are considered to form concurrently with the alkyl chain elongation, whereas the third double bond at the delta-7 position was predicted to form by an unsaturation reaction through a stable isotope labeling experiment (Kitamura et al. 2018). In fact, the gene encoding alkenone delta-7 desaturase (*alkenone desaturase 1*, *Akd1*) in *T. lutea* was experimentally characterized through gene overexpression, followed by the qualification of the ratio of C_37_ methyl alkenone with three double bonds to those with two double bonds (C_37:3_ to C_37:2_ ratio) (Endo et al. 2018). However, in the ecologically important haptophyte *G. huxleyi*, the alkenone metabolism-related genes have not been characterized. One factor hindering this characterization is the lack of applicable transformation techniques in *G. huxleyi.* Cai et al. (2021) reported the successful transformation in *G. huxleyi* using electroporation, but the colony formation was poor. Skeffington et al. (2020) reported the improvement of colony formation in *G. huxleyi*. Thus, the efficient transformation method in *G. huxleyi* might be developed in the near future. In other haptophytes, agrobacterium-mediated transformation was developed in *I. galbana*, *Isochrysis* sp. (Prasad et al. 2014), and *Pavlova lutheri* (Prasad 2017), and polyethylene glycol (PEG)-mediated transformation was developed in *Pleurochrysis carterae* (Endo et al. 2016) and *T. lutea* (Endo et al. 2018). Heterologous expression systems in yeast and cyanobacteria are often utilized for the characterization of fatty acid desaturases (Domergue et al. 2003). In the case of fatty acid desaturase evaluation, fatty acids as substrates of the desaturase are general molecules in the cells, and it is relatively easy to feed free fatty acids to the host cells. However, alkenones are not common molecules for organisms, and it is difficult for cells to incorporate hydrophobic and solid alkenones from the outside; therefore, utilization of a general heterologous expression system, such as in yeast or cyanobacteria, is not realistic for the characterization of alkenone desaturase or other alkenone metabolism-related genes. To overcome this issue, we introduced the putative alkenone desaturase gene from *G. huxleyi* into an alkenone-producing haptophyte for characterization. Among the transformable haptophytes, besides *G. huxleyi*, *Isochrysis* spp. and *T. lutea* are alkenone producers; therefore, we chose *T. lutea* as the host of the heterologous expression because the PEG-mediated transformation is relatively simple compared to agrobacterium-mediated transformation.

In the present study, we experimentally confirmed whether DesT in *G. huxleyi*, a homolog of *T. lutea*-derived Akd1, was the alkenone delta-7 desaturase for C_37_ methyl alkenone through heterologous expression in *T. lutea*. In addition, we reported that the possible tunnel required for the alkenone interaction with the reaction center existed in DesT. Our results pave the way for the characterization of the alkenone metabolism-related genes in *G. huxleyi* and other alkenone-producing haptophytes through the heterologous expression system in *T. lutea*.

## Materials and Methods

### Strains and Culture Conditions

The marine haptophyte *G. huxleyi* NIES-837 and *T. lutea* (formerly *Isochrysis* aff. *galbana* UTEX LB 2307) (Bendif et al. 2013) were used for the cloning of a putative homolog of alkenone delta-7 desaturase and as a host of heterologous gene expression, respectively. Modified Marine Art ESM medium (MA-ESM) (Danbara and Shiraiwa 1999) was used for cultivation. Cells of *G. huxleyi* and *T. lutea* were kept in 18 °C and 25 °C incubators, respectively, under constant illumination at 50–100 µmol photons m^−2^ s^−1^ without shaking.

### Construction of the Expression Vectors

The primers used in this study are listed in Supplementary Table S1. The putative alkenone delta-7 desaturase in *G. huxleyi* (*g454486*, *DesT*, GenBank ID: BBD52743.1) was searched in Protein BLAST using the amino acid sequence of Akd1 (GenBank ID: BBB21622.1), alkenone delta-7 desaturase in *T. lutea* as a query (Endo et al. 2018). Total RNA was extracted from approximately 4 × 10^7^ cells of *G. huxleyi* using TRIzol reagent (Thermo Fisher Scientific, Tokyo, Japan) and purified using a NucleoSpin RNA Kit (TaKaRa Bio, Shiga, Japan). cDNA was prepared using a PrimeScript RT Reagent Kit with gDNA eraser (TaKaRa Bio). The coding region of DesT was then amplified from the cDNA using PrimeSTAR HS DNA Polymerase (TaKaRa Bio) with EhDesT_N3flag_inf_Fw/Rv primers. In this PCR reaction, 9% (v/v, final concentration) dimethylsulfoxide (Fujifilm Wako Pure Chemical, Osaka, Japan) was added to the reaction mixture.

The plasmid structure and construction procedure are illustrated in Supplementary Figure S1. The pBlueScript SKII ( +) was first linearized by inverse PCR using pBSinv_Fw/Rv primers, and the insert fragment of the Lhcf17 promoter-driven *AphVII* expression cassette was then amplified using Lhcf17-Aph7_inf_Fw/Rv primers from a previously reported plasmid (Endo et al. 2018). Then, the linearized backbone plasmid and the insert fragment were fused using In-Fusion Snap Assembly Master Mix (TaKaRa Bio) to generate the pLhcf17p_Aph7 vector. The plasmid vector pLhcf17p_N3flag_LacZa_Aph7 was used as a template vector for the heterologous expression of alkenone delta-7 desaturase from *G. huxleyi*. In this vector, two expression cassettes, whose expressions were driven by the *lhcf17* promoter and terminator from *T. lutea*, were located in tandem. The downstream cassette was the expression cassette of the hygromycin resistance gene (*AphVII*) for the selection of the *T. lutea* transformant, and the upstream cassette was used to express the gene of interest (GOI). In the original template vector, the *LacZ* alpha fragment that was derived from pBlueScript SKII ( +) was inserted between the *lhcf17* promoter and the terminator of the GOI expression cassette, and this *LacZ* alpha cassette was flanked with *Eco*RV sites. Thus, the plasmid was first digested with *Eco*RV to remove the *LacZ* alpha fragment. Then, the digested plasmid and the PCR product (coding region of *DesT*) were respectively purified using a Cica Genius PCR & Gel Prep Kit (Kanto Chemical, Tokyo, Japan) and fused using In-Fusion Snap Assembly Master Mix (TaKaRa Bio) to generate the pLhcf17p_DesT_Aph7 vector. The sequence of the insert was confirmed by Sanger sequencing using a BigDye Terminator v3.1 Cycle Sequencing Kit (Applied Biosystems, Waltham, MA, USA).

### Preparation of the Protoplast and Transformation

*T. lutea* cells statically cultivated in 100 mL of MA-ESM medium in a 200-mL Erlenmeyer flask for 4–5 weeks were used for protoplast preparation and transformation as described previously (Endo et al. 2018). The pLhcf17p_DesT_Aph7 and pLhcf17p_Aph7 that contained only a single expression cassette of *Lhcf17* promoter-driven *AphVII* were introduced to *T. lutea* cells for the heterologous expression of DesT from *G. huxleyi* and mock transformation, respectively. The plasmid vectors were digested with *Sca*I for linearization and then precipitated using ethanol. After drying via vacuum centrifugation (MV-100, TOMY, Tokyo, Japan), the linearized vectors were dissolved in Milli-Q water (Merck Millipore, Tokyo, Japan). The concentration of the vector DNA was adjusted to approximately 1 µg µL^−1^, and 30 µL of the vector DNA solution (30 µg DNA) was used in a single transformation trial.

### Screening of Transformant

After 3 days of recovery culture in MA-ESM under continuous illumination (50–100 µmol photons m^−2^ s^−1^) at 25 °C, the cells were harvested via centrifugation (1380 × *g*, 5 min, 25 °C) and spread on a 1% agar-solidified half-concentration MA-ESM plate containing 2 mg mL^−1^ hygromycin B (Fujifilm Wako Pure Chemical). After 3–4 weeks of cultivation, the colonies were transferred to a 24-well plate containing 1 mL of liquid MA-ESM medium with 2 mg mL^−1^ hygromycin B and cultivated for 1 week. Then, the grown cell lines were transferred to a 50-mL disposable cell cultivation flask using a plug-sealed cup (VTC-F25P, AsOne, Osaka, Japan) containing 30 mL of liquid MA-ESM with 2 mg mL^−1^ hygromycin B.

Integration of the vectors into the genome was confirmed by PCR. The genomic DNA was extracted using a NucleoSpin Tissue Kit (TaKaRa Bio), and the partial regions of AphVII and DesT were amplified from the genomic DNA using AphVII_RT_Fw/Rv and DesT_RT_Fw/Rv primers, respectively. The expression of the transgenes was confirmed by reverse transcription PCR. Total RNA was extracted from the transformant using TRIzol reagent (Thermo Fisher Scientific) and purified using a NucleoSpin RNA Kit (TaKaRa Bio), and cDNA was prepared using a PrimeScript RT Reagent Kit with gDNA eraser (TaKaRa Bio). Then, the partial regions of AphVII and DesT were amplified from the cDNA using the same primers used for the genome PCR. GoTaq qPCR Master Mix (Promega, Madison, WI, USA) and Piko 96 Real-Time PCR System (Thermo Fisher Scientific) were used for the implementation of real-time RT-qPCR. In the RT-qPCR, specific primers for akd1 and heat shock protein 70 (Hsp70) that were reported in the previous study (Endo et al. 2018) were used for evaluation of expression level of akd1.

### Cultivation of Transformant

After 7–10 days of cultivation in the disposable cell cultivation flask, the cells were inoculated into a 100-mL cultivation tube containing 50 mL of liquid MA-ESM medium at an initial cell concentration of 1 × 10^5^ cells mL^−1^ and cultivated for 14 days under continuous illumination (100 µmol photons m^−2^ s^−1^) with 1% (v/v) CO_2_ bubbling at 30 °C. The cell densities were measured using a Thoma hemocytometer every 2 days, and the cultivated cells were harvested on the final day for alkenone analysis. We noted that the series of pre-cultivation (namely, colony pick-up and small-scale cultivation in 24-well plates and disposable flasks) was a continuous process, and the cells cultivated in this stepwise scaling up were immediately utilized for main cultivation to avoid attenuation of transgene expression by repeated subculture.

### Quantification of Alkenones

The cells harvested from 20 mL of culture were dried using a freeze-dryer (FDU-1100, TOKYO RIKAKIKAI, Tokyo, Japan). Then, the alkenone was extracted from the dried cells as described previously (Nakamura et al. 2016). Triacontane (C_30_H_62_, Fujifilm Wako Pure Chemical) was used as the internal standard. The extracted alkenone samples were applied to gas chromatography equipped with a flame ionization detector (GC-FID, GC-2014, Shimadzu, Kyoto, Japan) for quantification. Each alkenone and alkenoate species were separated using a VF-200 ms column (60 m × 0.25 mm × 0.10 µm, Agilent Technologies, Santa Clara, CA, USA) with a temperature setting described previously (Nakamura et al. 2016).

### Structural Prediction and Analysis

We used LocalColabFold version 1.5.2 (Mirdita et al. 2022), an implementation of AlphaFold 2 (Jumper et al. 2021), for protein structure prediction. For the tunnel analysis, we used the CAVER for PyMOL plugin version 3.0.3 with the following parameters (Chovancova et al. 2012): maximum probe radius of 0.6 Å, shell depth of 6 Å, shell radius of 2 Å, clustering threshold of 12 Å, and 12 approximating balls. The starting point of the tunnel was defined by the center of the geometry of the residues in the histidine box. The amino acid composition along the tunnel was calculated based on the probe balls, counting residues within a radius and 2-Å margin from each probe ball.

## Results and Discussion

### Nucleotide and Amino Acid Sequences of the Putative Alkenone Delta-7 Desaturase in G. huxleyi

**Fig. 1:**
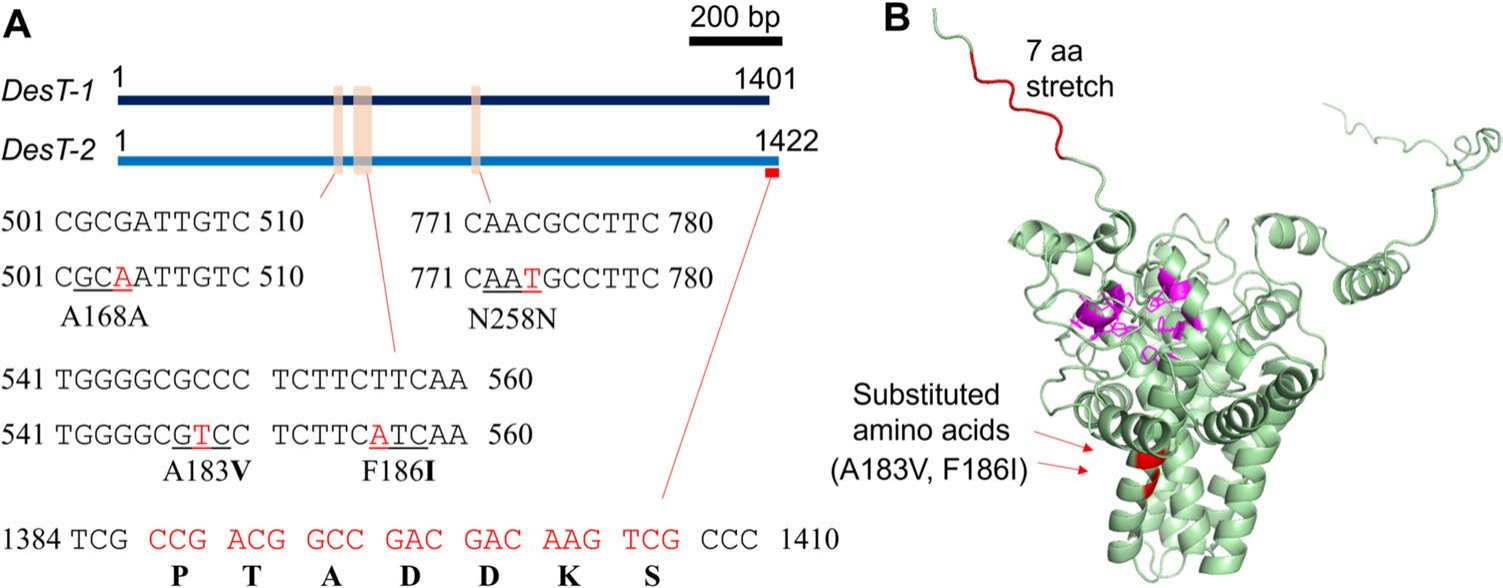
Graphical comparison of the DesT-1 and DesT-2 sequences. **A** Substituted nucleotides and amino acids in the coding region of DesT-2 compared with DesT-1. The beige rectangles indicate the varied regions between the nucleotide sequences of the *DesT-1* and *DesT-2* genes. The upper and lower nucleotide sequences correspond to the regions of the *DesT-1* and *DesT-2* genes, respectively. The red bar below *DesT-2* indicates the location of the insertion sequence. Red letters indicate the substituted and inserted nucleotides in the *DesT-2* gene. Bold characters indicate the substituted and inserted amino acids in DesT-2. **B** The protein structure of DesT-2 predicted by AlphaFold2. The red regions indicate the positions of the substituted amino acids and the inserted 7-amino-acid stretch at the C-terminal region in DesT-2. Magenta indicates the histidine box motifs that are conserved in fatty acid and alkenone desaturase

Through the vector construction, two types of coding sequence were cloned from the cDNA of *G. huxleyi* NIES-837. One sequence was identical to that in the genome sequence of *G. huxleyi* CCMP1516 (Read et al. 2013), whereas the other had four nucleotide substitutions (G504A, C548T, T556A, and C774T) and a 21-bp (7 amino acids) stretch at the C-terminal side of the protein (Fig. 1A). Among the eight *E. coli* clones analyzed, four had the former sequence and the other four had the latter sequence, suggesting that these could be transcribed products from different alleles. Thus, we designated the former and latter sequences “DesT-1” and “DesT-2,” respectively. Among the four nucleotide substitutions in DesT-2, two of them caused amino acid substitution (A183V and F186I, Fig. 1A). These amino acids were located in the transmembrane region, and the changes from alanine to valine at 183 aa and from phenylalanine to isoleucine at 186 aa were both substitutions of hydrophobic amino acids, suggesting that these amino acid substitutions did not drastically change the protein structure or function (Fig. 1B). The predicted protein structures of DesT-2 showed that the 7-amino-acid stretch at the C-terminal side was located at the relaxed end of the polypeptide (Fig. 1B). Thus, this stretch seemed not to affect the core protein structure, especially the histidine box motif that was conserved among the fatty acid and alkenone desaturases (Fig. 1B, shown in magenta).

The length of DesT-1 was 466 aa, slightly longer than that of Akd1 from *T. lutea* (446 aa). Amino acid sequence alignment using ClustalW revealed that the sequence identity between DesT-1 and Akd1 was 48.72%, and the sequences at the three histidine boxes showed high similarity (Fig. S2). According to a previous study, the first and third histidine box motifs are characteristic of Akd1 compared to other fatty acid desaturases (Endo et al. 2018), and DesT-1 conserved the same sequence features (namely, HRYXXH and HDHHH at the first and third histidine boxes, respectively) as alkenone desaturase (Fig. S2).

### Alkenone Composition of the Transformant

**Fig. 2:**
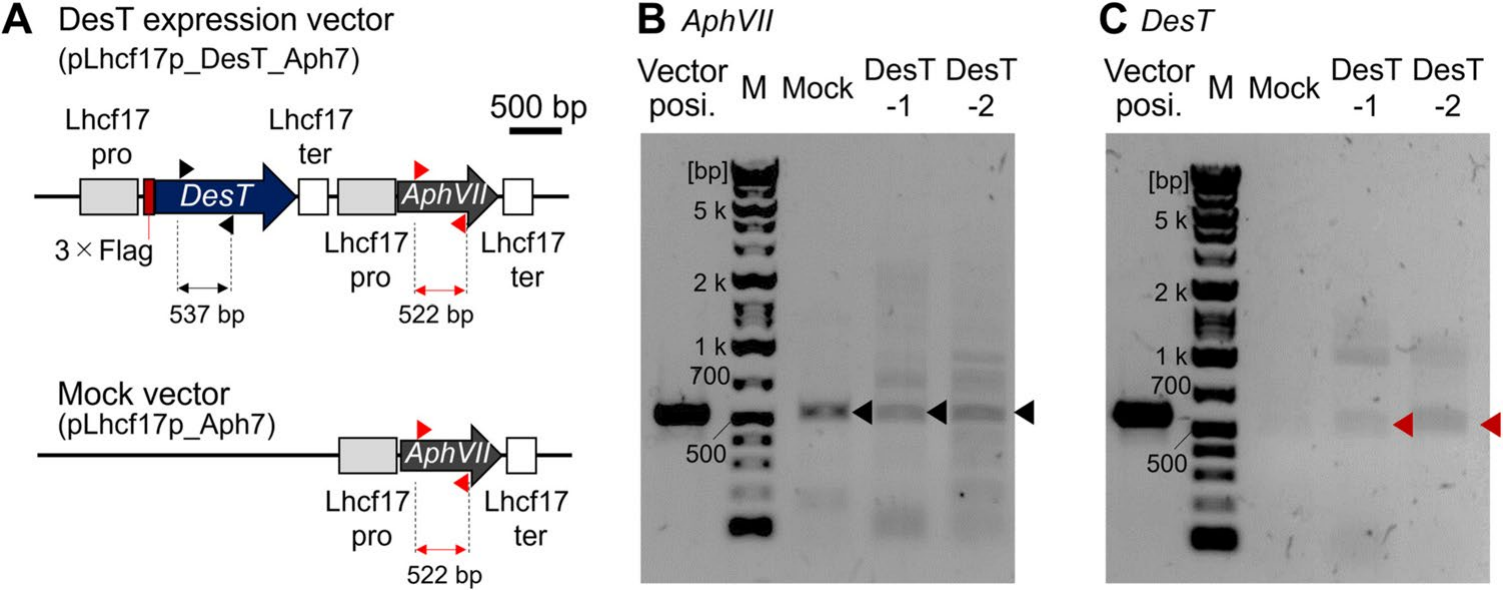
Structure of the expression cassettes and confirmation of transgene expression. **A** Structure of the DesT-expression vector (pLhcf17_DesT_Aph7) and a mock vector (pLhcf17_Aph7). Lhcf17 pro and ter indicate the promoter and terminator sequences of the genes for the light-harvesting complex *f*17 from *T. lutea*. Arrowheads indicate the annealing positions of the primers used for RT-PCR, and the double arrows indicate the sizes of the amplicons. **B**, **C** Gel images of agarose gel electrophoresis to confirm the expression of the *AphVII* and *DesT* genes in the mock vector and the DesT-1 and DesT-2 transformants through RT-PCR. Vector posi. indicates the positive control (samples amplified by PCR using the vector as a template). The black and red arrowheads in the gel images indicate the amplicons of the partial sequences of *AphVII* and *DesT*, respectively

**Fig. 3:**
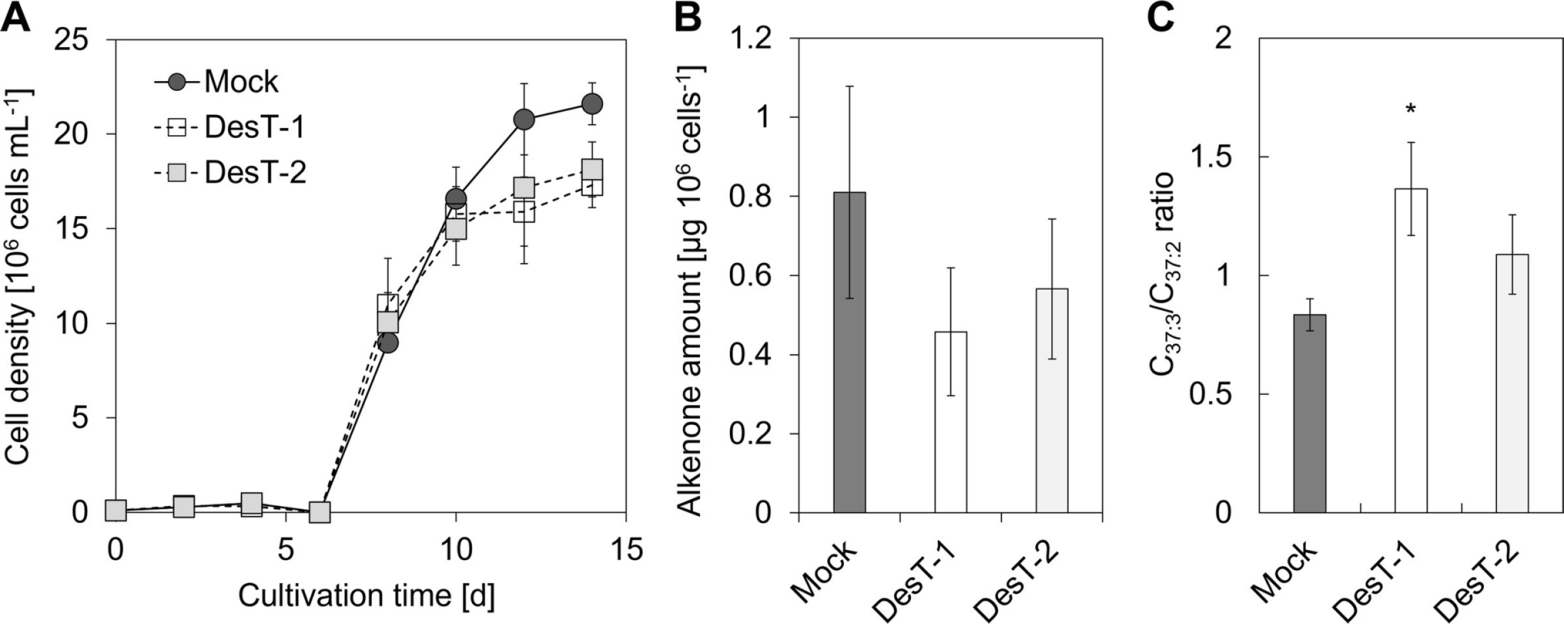
Growth and alkenone accumulation in the mock and DesT strains. **A** Growth in MA-ESM medium over 14 days. The solid and dashed lines indicate the growth curves of the mock and DesT strains, respectively. **B** Alkenone quantity per cell. **C** Ratio of C_37:3_ to C_37:2_ methyl alkenone. Statistical analysis was performed using Dunnett’s test (*P < 0.05, *n* = 3). Error bars indicate the standard deviation (*n* = 3)

The DesT heterologous expression vector and the mock vector carrying only the hygromycin resistance cassette (Fig. 2A) were introduced into *T. lutea* cells and screened in the presence of hygromycin. Integration of the vectors in the genome was evaluated through genome PCR (Fig. S3), and the expression of transgenes was confirmed through RT-PCR (Fig. 2B and C). Also, we confirmed that endogenous akd1 expression levels were not statistically different among the mock strain and the DesT-1 and DesT-2 transformants (Fig. S4). We cultivated the mock strain and the DesT-1 and DesT-2 transformants in MA-ESM medium for 14 days at 30 °C because the ratio of C_37:3_ to C_37:2_ methyl alkenone is lower at 30 °C compared to at 20–25 °C (Endo et al. 2018), and we expected the difference in the ratio of 3-unsaturated alkenone between the mock strain and the DesT transformants to be emphasized at 30 °C. The cell density of the mock strain was slightly higher than those of the DesT-1 and DesT-2 transformants, but the overall growth curves were not very different among the three strains (Fig. 3A). The total alkenone content was slightly decreased in the DesT-1 and DesT-2 transformants compared with the mock strain (Fig. 3B). This slight decrease in alkenone content was also observed in a previous report (Endo et al. 2018); however, the reason for this phenomenon is unknown. The ratio of C_37:3_ to C_37:2_ methyl alkenone in the DesT-1 transformant was significantly higher than that in the mock strain (Fig. 3C), suggesting that DesT-1 has the activity of delta-7 alkenone desaturase for C_37_ methyl alkenone as well as Akd1 in *T. lutea*. In the case of the DesT-2 strain, the ratio of C_37:3_ to C_37:2_ was also higher than that in the mock strain, but we did not detect a statistically significant difference (Fig. 3C).

### Protein Structure Prediction of DesT and Its Relationship with Alkenone

**Fig. 4:**
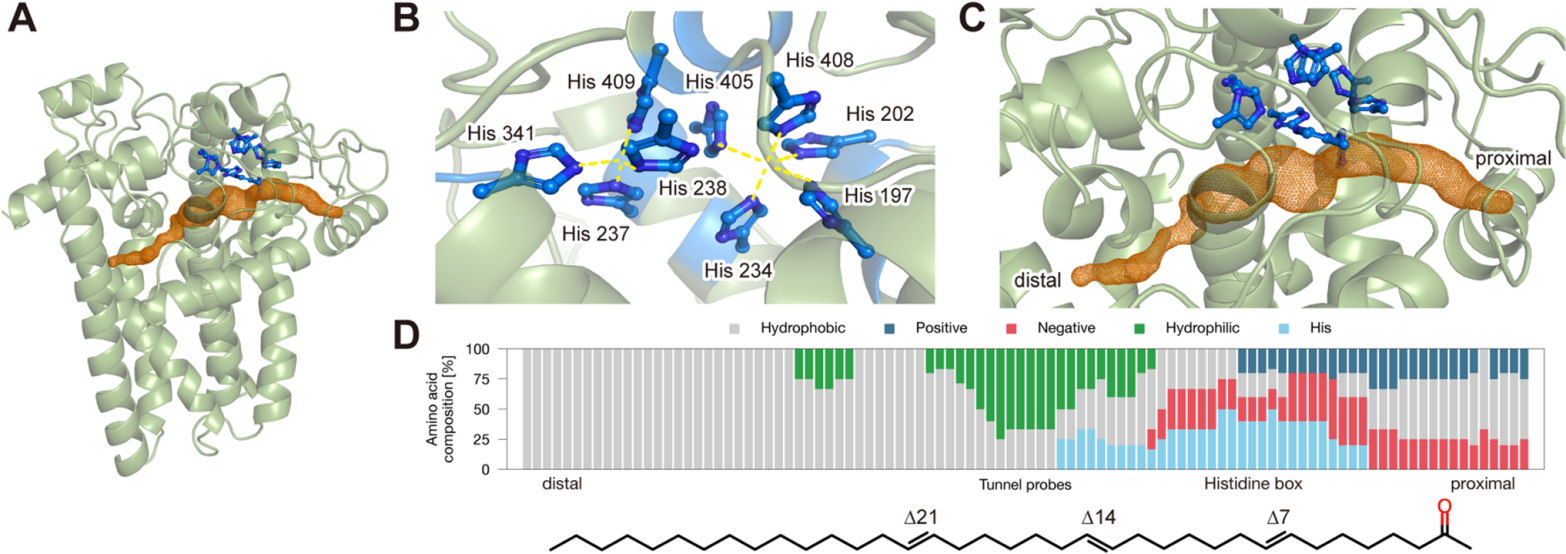
Structural prediction of DesT-1. **A** Predicted structure using AlphaFold 2 with the highest score. Unreliable terminal regions are not shown (see Fig. 1B). Blue sticks represent histidine residues in the histidine boxes. The detected cavity (alkenone tunnel) is shown as a brown mesh. **B** Enlarged view of the histidine boxes. **C** Enlarged view of the alkenone tunnel. **D** (Upper panel) Composition of the amino acid residues adjacent to the alkenone tunnel. In the cavity prediction, the shape of the tunnel was illustrated as a union of empty balls of various sizes, the radius of which was set as the maximum possible (Pavelka et al. 2016). The compositions of the amino acid residues that existed at the boundary of each empty ball (radius + 2 Å) are represented. Colors indicate the properties of the amino acids: hydrophobic (gray), positively charged (blue), negatively charged (red), hydrophilic (green), and histidine (light blue). (Lower panel) The structural image of the alkenone indicates the presumable orientation in the desaturase. The carbonyl group, which has a hydrophilic trend, is indicated in red

To examine the reaction mechanism of DesT as a delta-7 alkenone desaturase, we predicted its detailed protein structure from the amino acid sequence using AlphaFold 2 (Jumper et al. 2021). The predicted structures exhibited high predicted local distance difference test (pLDDT) scores (DesT-1: from 79.4 to 82.9; DesT-2: from 78.6 to 82.0), with five predicted structures in agreement (Fig. 4A, S5A, B). DesT-1 and DesT-2 exhibited nearly identical structures. Therefore, we focused on DesT-1 in the subsequent analyses. Both the N-terminal and C-terminal regions were predicted with low confidence, indicating disordered regions. The histidine boxes were found to consist of nine histidine residues, namely, His 197, 202, 234, 237, 238, 341, 405, 408, and 409 (Fig. 4B). The histidine box appeared to be composed of two parts, each capable of containing a coordinated metal (probably an Fe(II)/Fe(III) ion), as shown in previous studies (Reed et al. 2003, Nagao et al. 2019). Next, the predicted structure was analyzed using the cavity detection program CAVER (Chovancova et al. 2012). A long tunnel was found running alongside the histidine boxes (Fig. 4C). The length of the tunnel was 45 Å, which is sufficient for the length of the C_37_ alkenone (approximately 40 Å). The average radius was 0.97 Å, which is also consistent with the C-H bond distance (1.1 Å) (NIST Computational Chemistry Comparison and Benchmark Database 2022). The composition of the residues around the tunnel was analyzed (Fig. 4D). The residues neighboring the histidine boxes were hydrophilic or charged (proximal end), while the residues in the distal end were hydrophobic. This result may reflect the orientation of C_37_ alkenone, which has a hydrophilic carbonyl group. In this orientation, the ∆7 carbon is adjacent to the histidine box, supporting the alkenone analyses described above, indicating that DesT is a possible alkenone ∆7 desaturase of *G. huxleyi*.

## Conclusions

Characterization of the alkenone metabolism-related genes conserved in *G. huxleyi* is difficult due to the lack of applicable transformation methods. In the present study, we attempted to heterologously express the putative alkenone delta-7 desaturase gene, designated DesT, of *G. huxleyi* in the transformable and alkenone-producing haptophyte, *T. lutea*, to characterize its function. Through the construction of the expression vector, we cloned two types of cDNA for the DesT sequence from *E. huxleyi* NIES-837, which suggested that these were transcripts from different alleles of the *DesT* genes in the NIES-837 strain; thus, we designated those identical to the published gene sequence from *G. huxley*i CCMP1516 and the distinct one as DesT-1 and DesT-2, respectively. Although these allelic genes encoded almost similar but slightly varied proteins, the predicted structures resembled each other. Transformants expressing the hygromycin resistance gene only (mock strain), the *DesT-1* gene (DesT-1 transformant), and the *DesT-2* gene (DesT-2 transformant) were cultivated for 14 days at 30 °C. The ratio of C_37:3_ to C_37:2_ methyl alkenone was significantly higher in the DesT-1 transformant than in the mock strain, indicating that DesT-1 in *G. huxleyi* has the same alkenone delta-7 desaturase activity as Akd1 in *T. lutea*. This ratio was also higher in the DesT-2 transformant than in the mock strain, but the difference was not statistically significant. The predicted protein structure contained a tunnel probably required for the binding of the substrate alkenone molecules around the histidine boxes in DesT. The adjacent amino acids in this alkenone tunnel consisted of hydrophilic and charged amino acids at the proximal side and hydrophilic amino acids at the distal side from the histidine boxes, suggesting that the alkenone molecule (the hydrophilic carbonyl group of alkenone) is located at the proximal side. This configuration fits with the delta-7 position of alkenone to the histidine box region of DesT, indicating that DesT is likely to be an alkenone delta-7 desaturase. This is the first report of the experimental characterization of the alkenone metabolism-related gene conserved in *E. huxleyi*. The heterologous expression system using *T. lutea* could contribute to the further functional characterization of alkenone metabolism-related genes conserved in *G. huxleyi* and other haptophytes.

## Supplementary Information

Below is the link to the electronic supplementary material.

Supplementary file1 (PDF 1080 KB)

## Data Availability

All data generated or analyzed during this study are included in this published article and its supplementary information files.
