# Supplementary material for: Characterization of Delta-7 Alkenone Desaturase in Haptophyte Gephyrocapsa huxleyi Through Heterologous Expression in Tisochrysis lutea
Source: Mar Biotechnol (NY). 2025 Feb 8;27(1):44. doi: 10.1007/s10126-025-10427-y (PMC11807052; doi:10.1007/s10126-025-10427-y)
Supplement: Supplementary file 1 — Supplementary file1 (PDF 1080 KB) [file 10126_2025_10427_MOESM1_ESM.pdf]

Characterization of delta-7 alkenone desaturase in haptophyte *Gephyrocapsa huxleyi* through heterologous expression in *Tisochrysis lutea*

Marine Biotechnology

Kohei Yoneda[1,a,*], Chinatsu Kobayashi[2,a], Hiroya Araie[3], Rikuri Morita[4], Ryuhei Harada[4], Yasuteru Shigeta[4], Hirotoshi Endo[5], Yoshiaki Maeda[1], Iwane Suzuki[1]

[1]Institute of Life and Environmental Sciences, University of Tsukuba, 1-1-1 Tennodai, Tsukuba, Ibaraki 305-8572, Japan

[2]Graduate School of Science and Technology, University of Tsukuba, 1-1-1 Tennodai, Tsukuba, Ibaraki 305-8572, Japan

[3]Department of Biosciences, Kanto Gakuin University College of Science and Technology, Mutsuura-higashi, Kanazawa-Ku, Yokohama, Kanagawa 236-8501, Japan

[4]Center for Computational Sciences, University of Tsukuba, 1-1-1 Tennodai, Tsukuba, Ibaraki 305-8577, Japan

[5]National Institute of Technology, Tsuruoka College, 104 Sawada, Inooka, Tsuruoka, Yamagata 997-8511, Japan

[a]Equal contribution as first author, *Corresponding author

Corresponding author: Kohei Yoneda

Email: yoneda.kohei.gt@u.tsukuba.ac.jp (ORCID: 0000-0003-0734-0617)

Address: Institute of Life and Environmental Sciences, University of Tsukuba, 1-1-1 Tennodai, Tsukuba, Ibaraki 305-8572, Japan

Supplementary tables

Supplementary Table S1. Primers used in the present study.

Purpose	Primer name	Sequence
Vector construction	pBSinv_Fw	5'-GTCGGGAAACCTGTCGTG-3'
	pBSinv_Rv	5'-GTGGCACTTTTCGGGGAAA-3'
	Lhcf17-Aph_inf_Fw	5'-CCCGAAAAGTGCCACGGCGCAGTAGAAATGCACCG-3'
	Lhcf17-Aph7_inv_Rv	5'-GACAGGTTTCCCGACCGCGTCCTCGGCTGCAAAGG-3'
	EhDesT_N3flag_inf_Fw	5'-GACGACGACAAGGATATGTCTTCCCAGACCGTCGTG-3'
	EhDesT_N3flag_inf_Rv	5'-CCAGTAGCTTGCGATTTAGTCCGTCTTGGGCGACTTGTC-3'
Sanger sequencing	TisoN3flag_Fw	5'-ATGCTCGACTACAAGGACCA-3'
	EhDesT_seq363_Fw	5'-GCGCCCCATCCGGTAGCCC-3'
	EhDesT_seq429_Fw	5'-CGTGATCCAGTACCTGATGC-3'
	EhDesT_seq901_Fw	5'-CTCTTCCCGTGCCTCGA-3'
Genome PCR and RT-PCR	AphVII_RT_Fw	5'-TGATCAAGCTGTTCGGCGAG-3'
	AphVII_RT_Rv	5'-ACGAAGATGTTGGTCCCGTG-3'
	DesT_RT_Fw	5'-GTACGCCTACCTCGCCTTTT-3'
	DesT_RT_Rv	5'-ATGTGGTGAGGCACGTACTG-3'
Real-time RT-qPCR (Endo et al. 2018)	Akd1_qpcr_Fw	5'-AAGCTTTAGCTCGTCATGGATC-3'
	Akd1_qpcr_Rv	5'-GGCGATGAATCGATGGTC-3'
	Hsp70_qpcr_Fw	5'-GCTCCACTCGCATTCCCAAG-3'
	Hsp70_qpcr_Rv	5'-GTCTCCTCGCCACCCTCAC-3'

Supplementary Figures

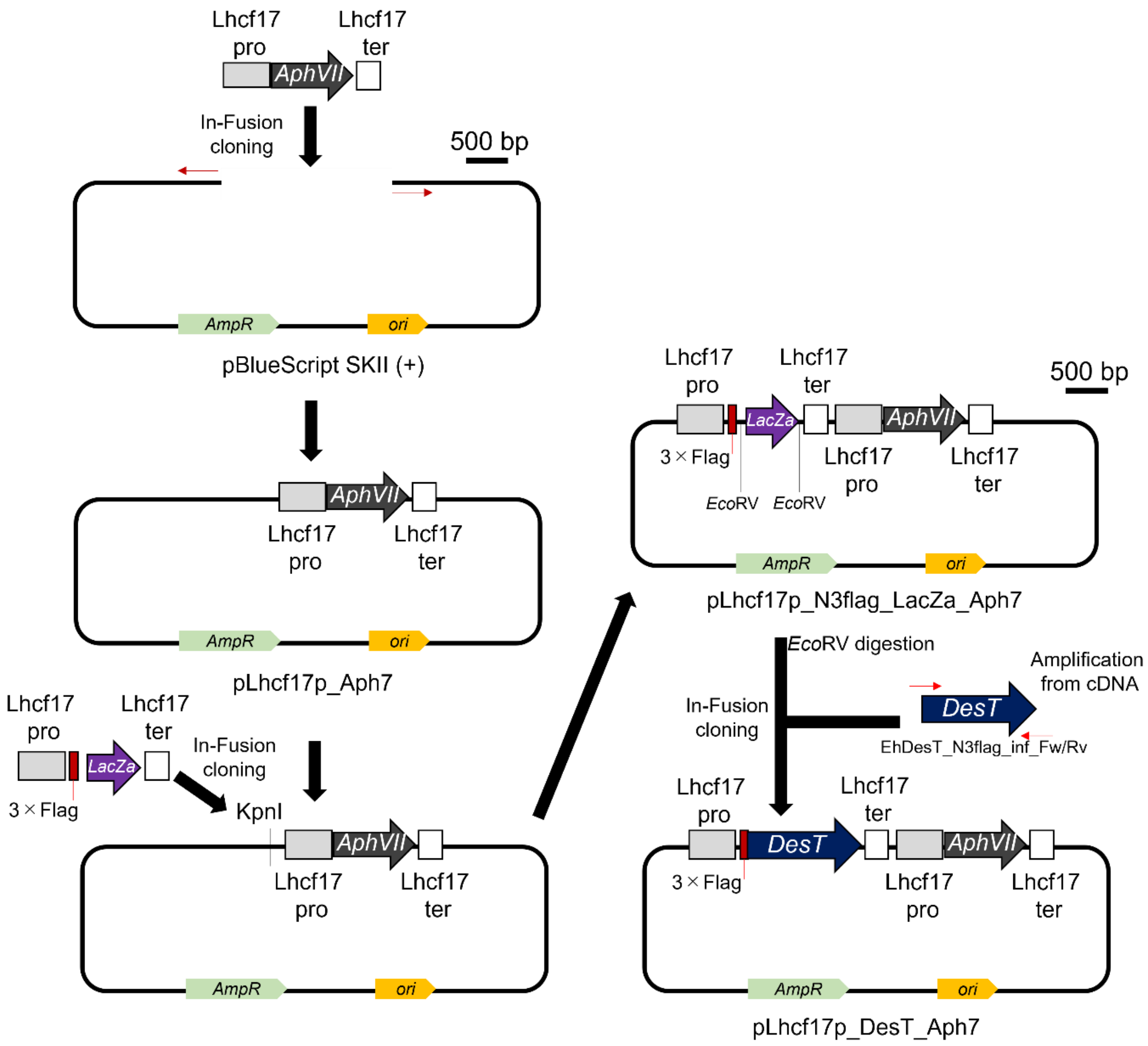

Fig. S1 Illustration of the procedure for the DesT expression vector construction

The *Lhcf17* promoter, terminator, and the *AphVII* coding sequence (PyAph7, formerly) were derived from a previous study (Endo et al. 2018).

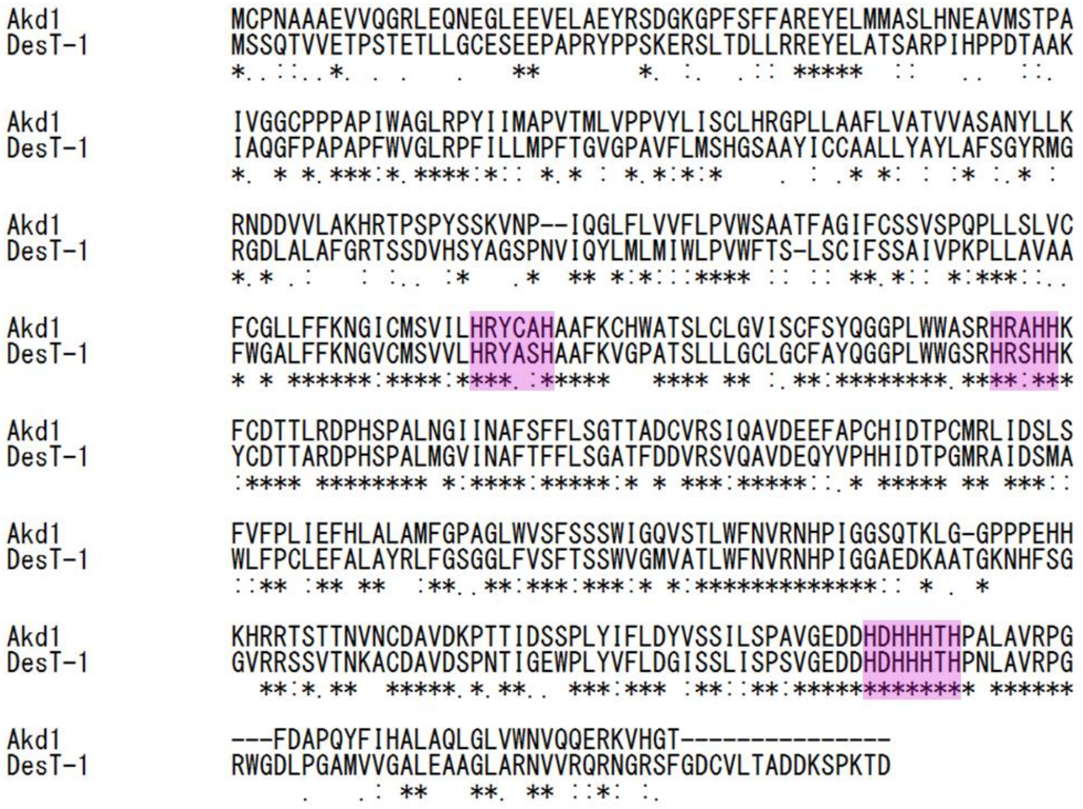

Fig. S2 Comparison of the amino acid sequences of Akd1 from *T. lutea* and DesT-1 from *G. huxleyi* Sequence alignment was performed using ClustalW. Areas highlighted in magenta indicate the histidine box motifs that are typically conserved in fatty acid and alkenone desaturase. The GenBank IDs of these alkenone desaturase are as follows: Akd1 (BBB21622.1) and DesT-1 (BBD52743.1).

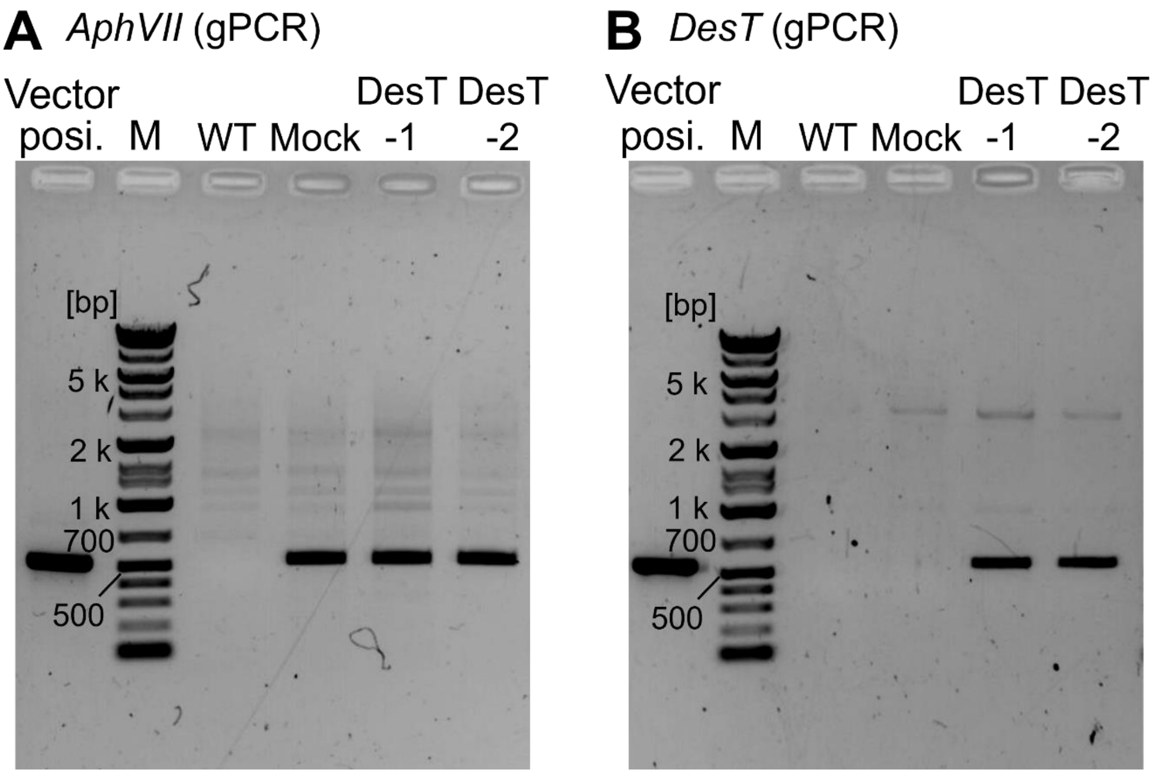

Fig. S3 Confirmation of the integration of expression vectors in genomic DNA

Gel images of agarose gel electrophoresis to confirm the integration of the *AphVII* (A) and *DesT* (B) vectors in the mock strain and the DesT-1 and DesT-2 transformants through genome PCR (gPCR). Vector posi. indicates the positive control (samples amplified by PCR using the vector as a template).

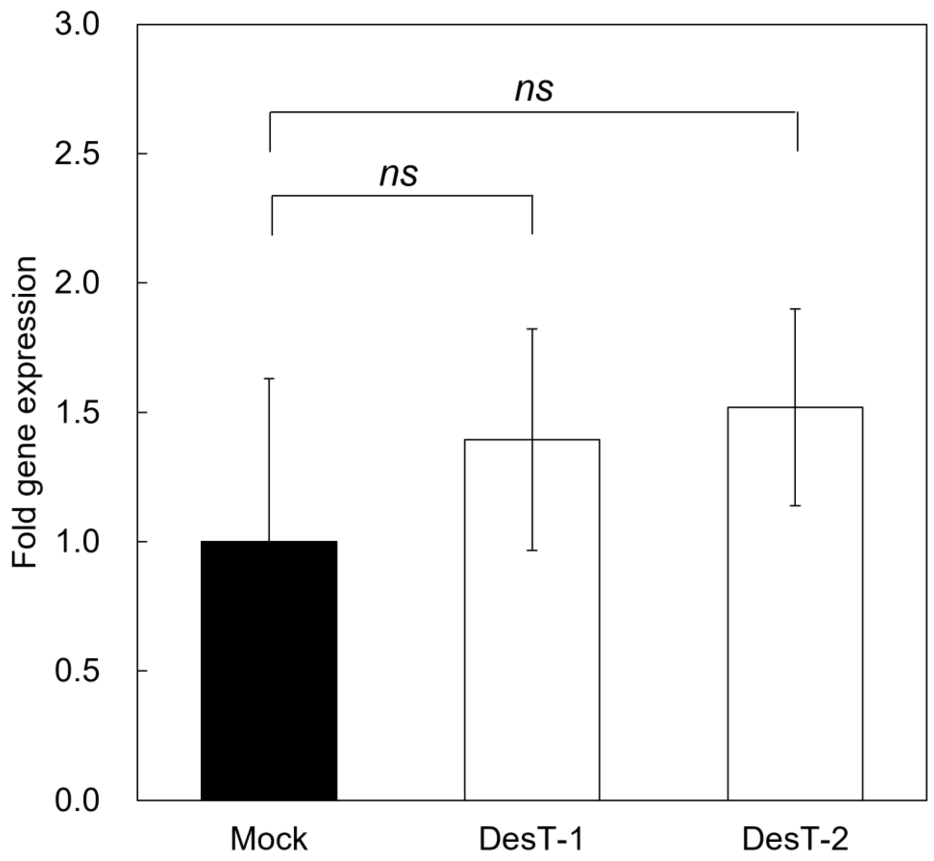

Fig. S4 Relative expression level of *akd1* in the mock strain and the DesT-1 and DesT-2 transformants. Real-time RT-qPCR and delta-delta Ct method were used to quantify the expression as described previously (Endo et al.2018). *Heat shock protein 70* (*Hsp70*) gene was used for normalization. Error bars indicate standard deviation of three technical replicate and the expression in the mock strain set as 1. Statical analysis was performed using Dunnett's test and *ns* indicates non-significant (P>0.05).

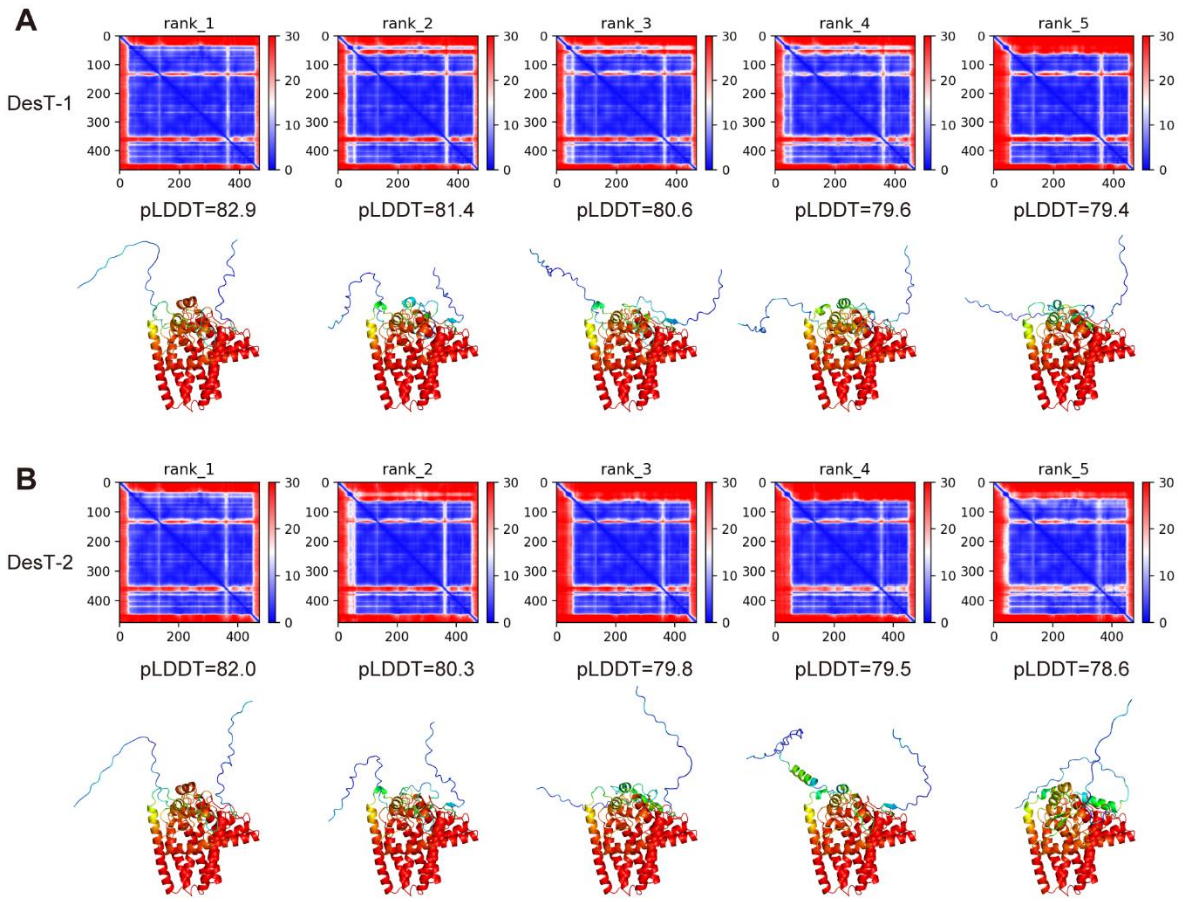

Fig. S5 Predicted structures of DesT-1 (A) and DesT-2 (B) using AlphaFold 2 with their predicted local difference distance test (pLDDT) scores

(Upper panel) Predicted alignment error for the top five prediction models. (Lower panel) Predicted structures of DesT corresponding to each rank. In the cartoon representation, the pLDDT scores of all residues range from low (blue) to high (red).

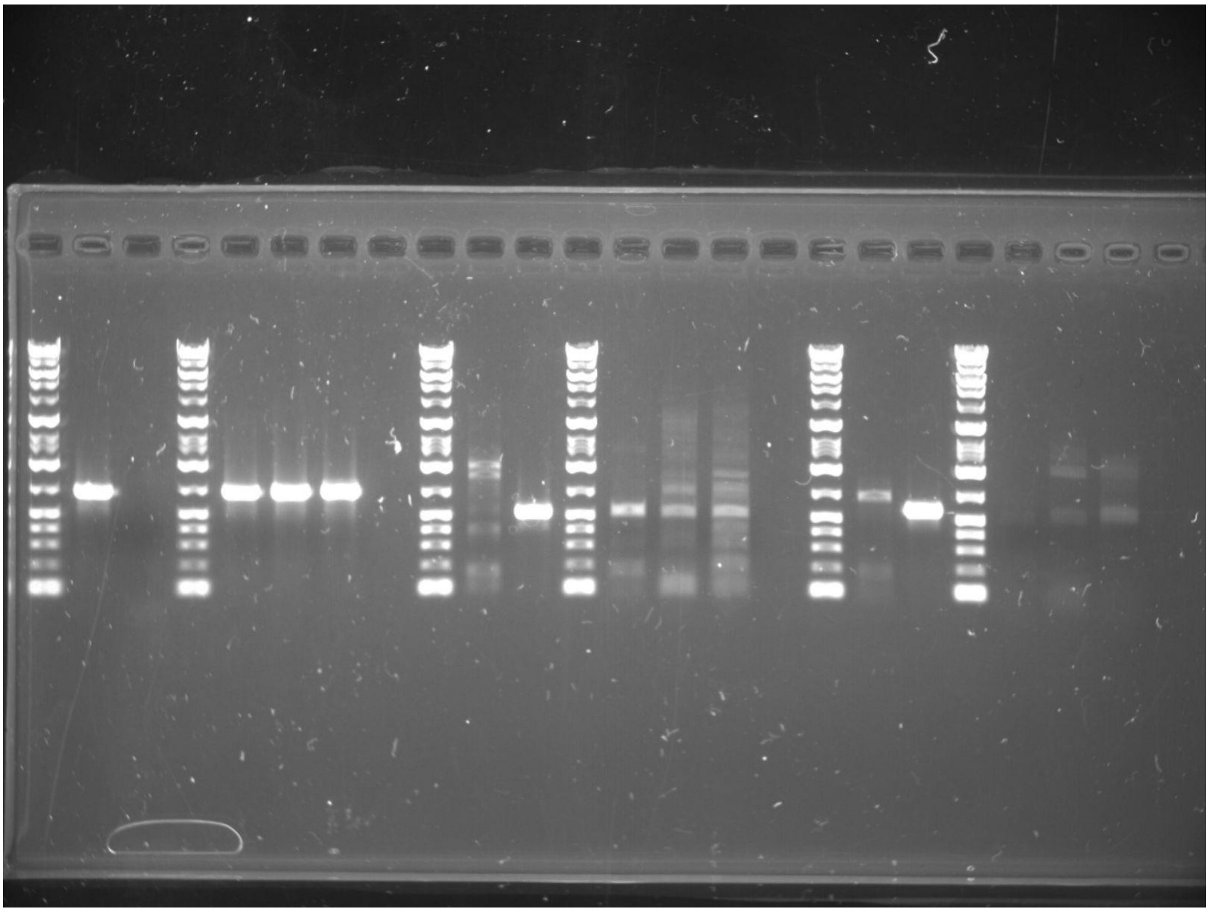

Fig. S6 Uncropped gel image of Fig. 2 B and C (RT-PCR)

Lane number (from left to right side) and combination of strain-amplicon: lane 1 (marker), 2 (WT, lhcf17), 3 (NTC, lhcf17), 4 (marker), 5 (mock, lhcf17), 6 (DesT-1, lhcf17), 7 (DesT-2, lhcf17), 8 (empty), 9 (maker), 10 (WT, aphVII), 11 (vector positive, aphVII), 12 (marker), 13 (mock, aphVII), 14 (DesT-1, aphVII), 15 (DesT-2, aphVII), 16 (empty), 17 (marker), 18 (WT, DesT), 19 (vector positive, DesT), 20 (marker), 21 (mock, DesT), 22 (DesT-1, DesT), 23 (DesT-2, DesT), 24 (empty)

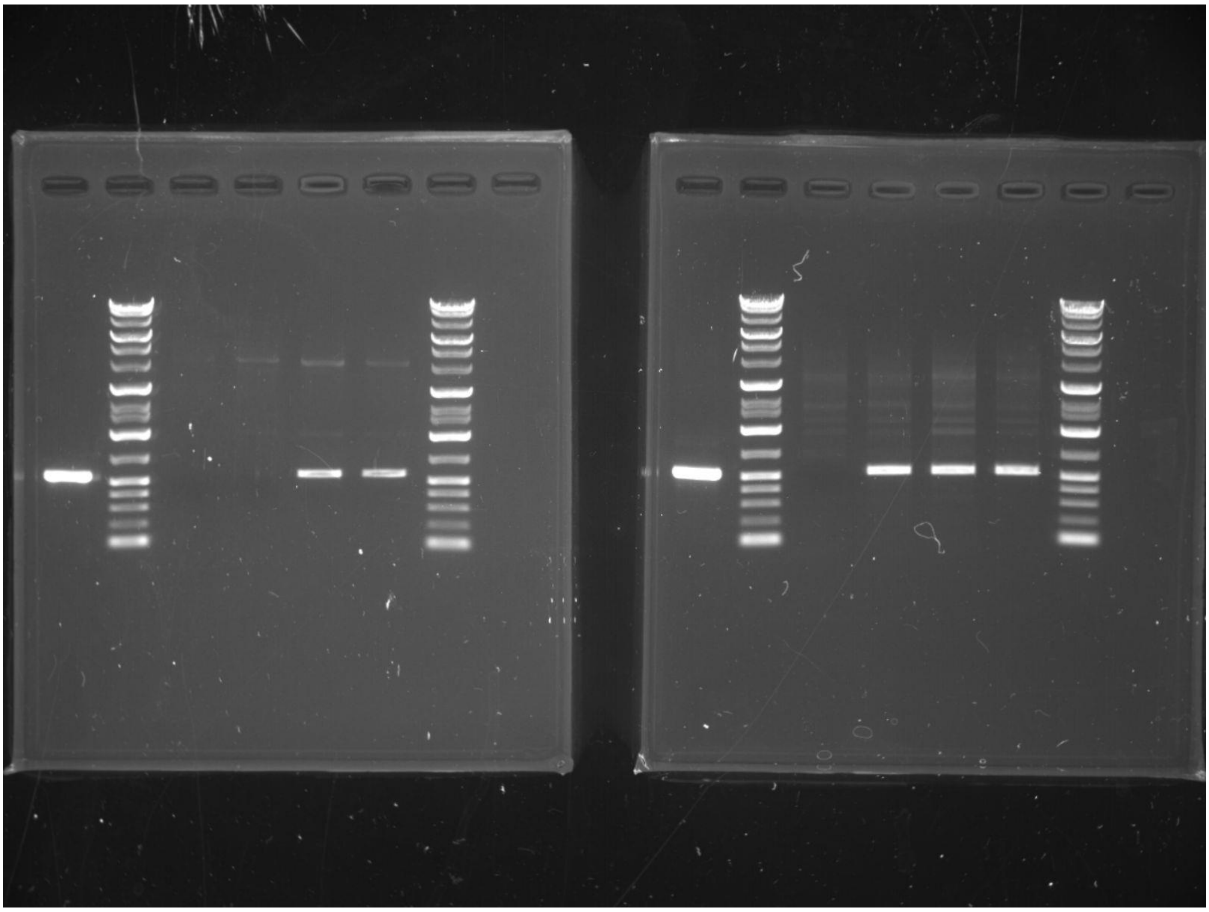

Fig. S7 Uncropped gel image of Fig. S3
